# The Therapeutic Nurse–Patient Relationship in Hemodialysis: A Pilot Mixed-Method Study on the Perceived Quality of Nurses’ Attitudes and Caring Behaviors

**DOI:** 10.3390/nursrep13030087

**Published:** 2023-07-20

**Authors:** Claudia Camedda, Gloria Bici, Camilla Elena Magi, Alice Guzzon, Yari Longobucco

**Affiliations:** 1 Department of Medical and Surgical Sciences, University of Bologna, 40138 Bologna, Italy; gloria.bici@studio.unibo.it (G.B.); alice.guzzon3@unibo.it (A.G.); 2 IRCCS Azienda Ospedaliero-Universitaria di Bologna, 40138 Bologna, Italy; 3 Department of Health Sciences, University of Florence, 50139 Florence, Italy; camillaelena.magi@unifi.it (C.E.M.); yari.longobucco@unifi.it (Y.L.)

**Keywords:** chronic kidney disease, therapeutic relationship, chronic diseases, nurse education, frailty

## Abstract

Chronic kidney disease affects many people around the world, leading those affected to replacement therapy such as hemodialysis. People who undergo hemodialysis generally undertake 2–3 treatments per week, lasting about 3–4 h each; patients spend many hours per week in contact with nurses, building a therapeutic relationship. The purpose of this work is to assess the quality of nurses’ perceived caring attitudes and behaviors and to determine their perceptions regarding the importance of the therapeutic relationship with the assisted patients. A self-reported questionnaire composed of three sections was administered to nurses; the first section included sociodemographic questions, the second the Caring Nurse–Patient Interaction Scale (CNPI-23), and the third part of the questionnaire was composed of open-ended questions investigating patients’ expectations according to nurses, the relevance of the therapeutic relationship on their work, and its effect on themselves and/or their own job satisfaction. Statistically significant correlations and trends have been observed between nurses’ sociodemographic data and the CNPI-23 items. In the clinical care area, nurses who have a post-basic degree or more years of experience feel more competent than those in other categories; in the relational care area, women tend to feel more competent than men. No correlations were found between the humanistic and comfort care areas. According to the results, the post-basic training of dialysis nurses and the adoption of organizational strategies that encourage nurse retention should be enhanced. This study was not registered.

## 1. Introduction

The number of people with chronic kidney disease is steadily increasing worldwide, affecting an estimated 843.6 million individuals in 2017 [1,2]. Factors contributing to this increase include improved survival in the general population, reduced mortality among dialysis patients, increased incidence of chronic kidney disease, and expanded acceptance criteria for renal replacement therapy [1,3]. According to the 2019 European Renal Association (ERA) Registries annual report, the European-wide incidence related to renal replacement therapy corresponded to 132 people per million population (pmp) [4]. In Italy, according to the 2019 Italian Registry of Dialysis and Transplantation (RIDT), out of the total national population of 45,607,293 people, there were 7327 patients undergoing replacement treatment, corresponding to an incidence of 162 people per million population (pmp) [4].

Hemodialysis patients usually visit dialysis centers two or three times a week for about four hours; here, nurses are responsible for the implementation of the treatment, following the patients from the beginning to the end of each session, and this can significantly influence the person’s private and professional life [5]. The relationship established between the nurse and the person on dialysis aims to achieve specific health-enhancing and therapy-supportive behaviors in the latter [6]. The therapeutic relationship between the nurse and patient seems to have a significant importance for people with end-stage renal disease who undergo repeated hemodialysis treatments [7]. The concept of the “therapeutic relationship” has been the focus of nursing theorists since after the Second World War until the 1990s [8]. Hildegard Peplau’s work *A Conceptual Frame of Reference for Psychodynamic Nursing* is considered to be the first theoretical formulation of nursing care as a therapeutic relationship; although Peplau did not refer the periphrasis “therapeutic relationship” to nursing care, she used the term “therapeutic” to refer to nursing care effects, and through the “relationship”, the nurse will obtain a therapeutic effect. Peplau describes nursing care as “a meaningful, therapeutic interpersonal process” [8]. According to the Registered Nurses Association of Ontario, the therapeutic relationship is based on the interpersonal process that occurs between the nurse and the patient; it is an intentional, goal-oriented relationship aimed at promoting the best interests of and the best outcome for the patient [9]. In a recent conceptual analysis, the therapeutic relationship was considered one of the attributes of quality nursing care [10]. A 2001 study collected experiences from dialysis nurses regarding the importance of the therapeutic nurse–patient relationship and the benefits it can bring; it reported that better listening skills toward the persons being cared for, focusing on their life and history, allows for effective relationship building and helps to obtain useful information about the patients’ health status [11]. The nurses noted the importance of not rejecting emotions, even if negative, as in the case of angry or frustrated patients; they suggested encouraging the expression of this feeling in order to direct the patient’s attention to the goal they have to achieve instead of denying the presence of it [11,12]. Thus, it emerges how an effective therapeutic relationship between nurse and patient can also bring benefits to health care professionals who reported how they have been encouraged by their patients with whom they shared their feelings during a hard period of their private life [11,13]. Ignoring the relational aspects of care may lead to dehumanizing practices toward patients, including de-individualization, nurse-assisted person dissimilarity, mechanization, and reduced empathy [14]. The potential consequences of uncaring may make the people who experienced it feel humiliated, powerless, out of control, hopeless, vulnerable, frightened, and alienated, and may lead them to relive unpleasant memories [13,15]. Mechanization and empathy suppression produce feelings of mistrust, suspicion, and misunderstanding in people, causing them a delay in physical recovery, which can result in the prolongation of hospitalization itself [14]. The consequences of adopting an indifferent attitude can lead health professionals to perform a purely technical assistance with an attrition risk toward patients and a feeling of fear and depression [15]. Furthermore, many professionals experience the phenomenon of burnout, which is a prolonged response to emotional and interpersonal stressors, characterized by emotional exhaustion, depersonalization, and lack of social fulfillment [16,17]. For patients with chronic diseases, the effectiveness of care is strongly influenced by the quality of nurse–patient communication [6]. Technical and practical skills have become a pivotal aspect of nursing practice, sometimes at the expense of the relational aspect [18]. In Marta Hreńczuk’s [7] study carried out in Poland, the aspect of the therapeutic nurse–patient relationship was deeply explored with a focus on long-term hemodialysis patients; the guiding purpose of the study was to identify hemodialysis patients’ perceptions of the therapeutic relationship with nurses and its importance. The results reported on the meaning that patients give to the therapeutic relationship and its main components, such as empathy, mutual trust, respect, the openness of both parties, joint verbal contact, friendliness, nonverbal contact, sense of security, understanding, and acceptance; then, it noted the positive feelings linked to this relationship which are considered important in renal replacement therapy [7]. Participants who reported the relevance of the nurse in the nurse–patient relationship also said that it gave them a feeling of being understood, giving them the strength to fight the disease, restoring hope, and helping them develop the inner ability to understand their own problems by looking at them through another person’s eyes, thus aiding to control feelings of helplessness while reinforcing the positive aspects of health behaviors [7]. Regarding the ability of the therapeutic relationship to influence the effects of therapy, the patients interviewed reported that the therapeutic nurse–patient relationship influenced their sense of security, helped them cope with difficult times, and improved the quality of health care they received; less frequently, they felt it helped them accept the illness and therapy, and increased the effectiveness of treatment. Most respondents in the Polish study believed that the nurse’s contact with the patient in a dialysis center was therapeutic [7].

The purpose of this pilot study is to assess the quality of nurses’ perceived caring attitudes and behaviors and to determine their perceptions regarding the importance of the therapeutic relationship with patients undergoing hemodialysis, while testing an adoptable tool for a subsequent multicentric study.

## 2. Materials and Methods

The study was conducted in January and February 2022 at the Dialysis Unit of Istituto di Ricovero e Cura a Carattere Scientifico (IRCCS) Policlinico Sant’Orsola, with the approval of the Bioethics Committee of the University of Bologna (Prot. n. 0322422 of 27/12/2021). The design of this pilot study is a mixed-method with a descriptive, cross-sectional, quantitative part and an exploratory, descriptive, qualitative part. Due to the pilot nature of this study, we did not perform any kind of sample size estimation; we adopted convenience sampling, consisting of nurses working in the abovementioned unit willing to participate in the study, recruited through an introductory conversation carried out before obtaining informed consent. All nurses who gave consent and signed the information form were included. To assess the perceived quality of attitudes and caring behaviors of dialysis nurses, a self-reported questionnaire consisting of 3 parts was developed; the first part consists of 6 sociodemographic questions investigating age, gender, educational qualifications, post-basic education, and length of service in the profession and dialysis setting. The second part consists of the self-assessment scale, adapted from the Caring Nurse–Patient Interaction Scale (CNPI-23 nurse version) [19]; it is a 5-point Likert scale composed of 23 questions divided into four areas: clinical care, consisting of 9 questions; relational care, consisting of 7 questions; humanistic care, consisting of 4 questions; and, finally, comforting care, consisting of 3 questions. The third part of the questionnaire contains three open-ended questions investigating, respectively, what nurses believe the patients they care for expect, whether they consider the therapeutic relationship relevant to their work, and whether they believe the therapeutic relationship may have effects on themselves and/or their own job satisfaction. The anonymity of the nurses involved was ensured to reduce potential bias. A descriptive and inferential analysis was conducted, after an assessment of the normality of the distribution with a Shapiro–Wilk test. Data were described by absolute frequency and the percentage or mean and standard deviation when appropriate. The reliability of the adopted version of the CNPI-23 tool was verified with Cronbach’s alpha coefficient. In order to assess the construct validity, a statistical analysis was performed using a Spearman’s correlation coefficient test; a nonparametric test was chosen because of the expected limited sample size. Data collected from open-ended questions were examined through content analysis, which allowed the recognition of items with the highest frequency of occurrence, and descriptive phenomenological analysis, which allowed inferences of categories representative of nurses’ perceptions. A probability level of <0.05 was considered significant for all the analyses. All the analyses were conducted with Stata 13^®^. The reporting of this study is compliant with the Strengthening the Reporting of Observational Studies in Epidemiology (STROBE) reporting guidelines for observational studies.

## 3. Results

The questionnaire developed for this study was proposed to a total of twenty-six nurses, employed in two dialysis departments of the same hospital; twenty-three agreed to participate and complete the survey. The first part of the questionnaire consists of six sociodemographic questions whose main data are shown in Table 1; in particular, the age of the sample examined shows a majority of nurses in the age range of 46 to 55 years (35%), with an average age of 42.17 ± 10.08 years. As their basic qualification, 65% have a bachelor’s degree, while the remaining 35% hold a bachelor’s degree (Before DM 509/99). Regarding professional experience, participants were asked about their years of working in hemodialysis and years of working as a nurse, including different settings for hemodialysis. The average number of working years in hemodialysis is 10.61 ± 8.73 and the average of working years as a nurse, including other settings, is 18.04 ± 9.80 years.

The second part of the questionnaire is the Caring Nurse–Patient Interaction Scale, summarized in Table 2; this is an instrument for assessing the quality of caring attitudes and behaviors perceived by nurses, consisting of a 5-point Likert scale (not at all competent, not very competent, moderately competent, very competent, and extremely competent) and composed of 23 items divided into four sections, respectively, clinical care, relational care, humanistic care, and comforting care. Regarding clinical care, investigated in the first nine items, nurses feel most competent in activities such as checking the outcome of patients after administering medications to relieve any reported symptoms, correctly administering treatments, knowing how to use specialized equipment, and carefully monitoring patients’ health conditions. Next, we have the ability to know what activities to perform at times when timeliness is required and showing skill and competence in how to intervene with patients: helping patients with care that they cannot manage on their own and providing them with guidance and means to treat or prevent some side effects of drugs or treatments. The item with the least feedback from nurses is the ability to provide patients with the opportunity to practice self-care. In relational care, nurses self-perceive themselves to be more competent in trying to identify with patients the consequences of their health behaviors, helping them to see things from another point of view, and helping them to recognize ways to effectively solve their problems. Next, we have the ability to help patients explore the meaning they give to their health condition, seek balance in their lives, and help them explore what is important in their lives. As the last item, there is the ability to help patients clarify what things they would like significant people to provide for them. The item with the highest feedback from nurses in humanistic care is treating patients as complete individuals, showing interest in more than just their health problem. Next, we have the ability to emphasize the efforts of the patients and knowing how to adopt a nonjudgmental attitude, as the last item is the ability to urge patients to be confident when appropriate. The last three items related to comforting care are the ability to carry out treatments or administer medications according to set schedules, respecting patients’ privacy, and taking into account their basic needs.

Regarding the reliability of the CNPI-23 tool, the Cronbach’s alpha coefficient of the 23 questions was 0.945. Table 3 shows the results of the construct validity testing, showing Spearman’s correlation coefficient values with corresponding “*p*” values in correspondence to the associations between the sociodemographic data and the items of the Caring Nurse–Patient Interaction Scale; because the sample size was small, we decided to report in the table both the variables that correlate with each other in a statistically significant way and the variables that show a correlation trend. As can be seen from the table, nurses who feel more competent in administering treatments tend to have more post-basic degrees than their other colleagues; while in the use of specialized equipment, there is a statistically significant correlation with years of experience in hemodialysis, so as the latter figure increases, more professionals will feel competent in the use of equipment. Regarding providing patients with the indications and means to treat or prevent some side effects of drugs or treatments, nurses with a higher age and those with more post-basic degrees show more competence. In situations in which it is necessary to act quickly, the professionals who show more confidence are those with greater age, compared to younger colleagues, while we observe statistically significant correlations with years of experience in both hemodialysis and nursing in general; thus, as years of experience increase, the sense of competence in situations in which it is necessary to act quickly increases. Similarly, professionals with more years of work in hemodialysis show a greater tendency to exhibit skill and competence in how to intervene with patients. A statistically significant correlation is observed between the ability to closely monitor patients’ health conditions and professionals’ post-basic educational qualifications, i.e., nurses with more post-basic educational qualifications consider themselves more competent in this field. Similar to this item, there is a trend that correlates the ability to provide patients with opportunities to practice self-care with the post-basic educational qualification of professionals. The ability to help patients recognize the means of effectively solving their problems and that of helping them see things from a different point of view are correlated through a trend to the gender of nurses, meaning that female professionals consider themselves more competent in these fields than their opposite-sex colleagues.

The third part of the questionnaire administered to nurses consisted of three exploratory open-ended questions, shown below:

- what do you think your patients expect from the nurses who care for them?
- do you believe that the therapeutic nurse–patient relationship is important and/or relevant in your work?
- do you believe that the nurse–patient relationship can also affect you as a person and/or your personal satisfaction?

Regarding the first question, the categories deduced from the analysis of the open-ended responses are as follows: Professional skills, Relational skills, Caring for the person, Elements of the relationship, and Dehumanization. The categories that emerged in the responses to the second open-ended question are the following: Important element of quality of work, Human relationship characterizes the professional, Supports nurses and patients in the care journey, Fundamental in chronicity, and Conditioned by reciprocity. Regarding the third question, the categories manifested are as follows: Proportional professional and personal satisfaction, Feeling professionally involved, Feeling personally involved, Seesaw satisfaction, and Dehumanization. The summary analysis of the responses containing the categories synthesized from the text, with their descriptive labels and respective occurrences, are shown in Table 4.

## 4. Discussion

The purpose of this study was to assess the quality of caring attitudes and behaviors perceived by dialysis nurses through the administration of the Caring Nurse–Patient Interaction Scale and to investigate their perceptions about the importance of the therapeutic relationship with patients undergoing hemodialysis.

From the results obtained, several correlations characterized by either trends or statistical significance can be observed; however, these clusters are mainly in the domain of clinical care and less in the relationship care area. The domains of humanistic care and comforting care report no correlation with the sociodemographic characteristics of the participants.

In the clinical care domain, possession of postbaccalaureate degrees makes nurses feel more aware about different abilities such as treatment administration, providing direction and means to treat or prevent the certain side effects of medications or treatments, closely monitoring patients’ health conditions, and supporting patients’ self-care practice. Our findings are confirmed by some other studies that suggest a positive correlation between training and caring attitudes and behaviors [20]. Because caring practices tend to fade over time, regardless of the nurse’s level of education [21], educational reinforcement for established practitioners by creating educational pathways that succeed in evoking feelings of caring in nurses might also be relevant.

Practitioners with more years of experience as a nurse and as a hemodialysis nurse feel more competent than their younger colleagues regarding the ability to act quickly when needed; more experience in hemodialysis also makes nurses more confident in using specialized equipment and showing skill and competence in how to intervene with patients. Numerous studies have established a correlation between nurse retention and care outcomes [22,23], including in the hemodialysis unit where higher turnover rates increase costs at discharge, unplanned re-hospitalizations, and patient mortality [22]. For these reasons, it might be useful to adopt organizational strategies that promote nurse retention and reduce the phenomenon of “pathological turnover”, i.e., the hemorrhaging of staff caused by job dissatisfaction, burnout, and the leadership style of managers [22].

In relational care, a predisposition emerged in female nurses, compared with their male colleagues, in feeling more able to help patients recognize ways to effectively solve their problems and to help them see things from a different point of view.

In the last section of the questionnaire, hemodialysis nurses were able to express their opinions about what patients expect from them, about the importance and relevance they attach to the therapeutic relationship in their work, and about the effects that the relationship has on their own personal and job satisfaction.

Regarding the first question, the main elements that emerged from the professionals’ opinions are technical skills and professionalism; this result suggests a thought focused on the quality of the technical act delivered by nurses. Reflections also emerged referring to relational skills, emphasizing how important it is to take care of the relationship itself with the patients, empathizing, having consideration toward the patients, and understanding toward the relevance of their requests, working on the time of care. Other suggestions emerging from the professionals’ responses report the proper elements of the relationship, such as empathy, understanding, trust, listening, reassurance, helpfulness, kindness, enacting nonjudgmental attitudes, dialogue, respect, politeness, and humanity. However, elements recalling the phenomenon of dehumanization such as pandering, complacency, subservience, and professional automatism also emerged.

In the second question, as many as 16 nurses among those interviewed focused on the relevance of the relationship in their work, with statements highlighting how an empathic relationship improves the quality of the work itself, such as “choosing nursing means choosing to be human”. Further suggestions from professionals also focused on the purpose of the therapeutic relationship, defining it as facilitating both the nurses, providing them with safety, and the patients themselves in the care pathway; according to nurses, the relationship acts as a mediator in achieving health goals, increasing engagement in health education, and supporting dialysis through mutual understanding and respect as its own elements.

The suggestions given by professionals draw the attention to those reported by hemodialysis patients interviewed in a Polish study, who said that the therapeutic relationship with nurses gives them confidence, helps them cope with difficult moments, helps them accept their illness and therapy, and increases the effectiveness of therapy and the quality of care [7].

Finally, in the last question, considerations of job and personal satisfaction emerged; some of the respondents define these two elements as proportional to each other, and they relate them. Starting with job satisfaction, the elements most highlighted by nurses are gratification, a sense of appreciation, joy at achieving professional milestones, and a sense of sadness at the onset of any complications. Personal satisfaction is connected to a personal, intimate, and lasting relationship with patients; the ability to satisfy people’s needs makes the nurses feel personal satisfaction and creates a mutual-exchange relationship. Other reflections reintroduce the theme of the empathic relationship, which complete the profile, together with specialized skills, with feelings of gratification in providing well-being, with the ability to become attached to chronic patients and form relationships by providing happiness. Not all responses to this question were characterized by clear thinking but some showed fluctuating satisfaction; moreover, elements characterizing dehumanization contained in the words “after so many years of working with chronic patients you must be able to hear without listening” also emerged. However, in spite of rare negative reflections, most of the nurses involved consider the therapeutic relationship an essential element of caring for patients and recognize its positive effects on their health outcomes.

This pilot study has some limitations. First of all, the sample size was quite small, even if it reached most of the nurses of the dialysis departments involved (23/26). Nevertheless, the findings can be representative of only one highly specialized hospital in northern Italy; several aspects could have influenced the results, such as the organizational culture of the hospital, the philosophy of the operating unit, the practices and habits of the staff in approaching patients, and the peculiarities of the setting itself. Furthermore, the tool used for data collection also represents a limitation, as it is not a validated self-reported tool, with answers expressed using a 5-point Likert scale, which could induce respondents to passively choose the central value of 3 in the case of indecision, rather than trying to lean to one side or the other of the scale, contrary to what would happen, for example, with a 4-point Likert scale. Nevertheless, a few psychometric characteristics of the tool could be addressed, and for further investigations, this tool should be validated in the Italian context. The authors believe that extending the research to other centers throughout the country could provide further suggestions and more representative results.

## 5. Conclusions

People with chronic kidney disease who resort to hemodialysis undergo about 2–3 treatments per week lasting about 3-4 h each; patients spend many hours per week in dialysis centers in contact, most of the time, with nurses, with whom they establish a relationship that acquires a “therapeutic” connotation at the moment when it promotes the achievement of health outcomes in the patients. Conducting reflections on the topic of the therapeutic nurse-assisted relationship is important to avoid as much as possible any dehumanizing behaviors, that is, those attitudes that deprive the person of his or her identity, reducing him or her simply in the terms of his or her illness; such behaviors could have effects, including psychological ones, on the person’s physical health, compromising the care pathway and affecting treatment outcomes. Having a postbaccalaureate degree seems to be a factor that makes professionals feel more competent in many aspects of the therapeutic relationship. For this reason, it would be appropriate to enhance postbaccalaureate education for nurses and educational reinforcements for established professionals by creating educational pathways that succeed in evoking feelings of caring in nurses. Since many competencies have been shown to be closely related to nurses’ years of experience in the field of hemodialysis, it might be useful to adopt organizational strategies that promote nurse retention and reduce the phenomenon of “pathological turnover”. One possible viable solution in the immediate term is to ensure adequate staffing with an even skill mix across shifts.

## Figures and Tables

**Table 1:** Sociodemographic data.

Data		N (%)	Mean (SD)
**Age**	25–35 y	6 (26)	42.17 (10.08)
	36–45 y	7 (30%)	
	46–55 y	8 (35%)	
	56–65 y	2 (9%)	
**Gender**	Woman	15 (65%)	
	Man	8 (35%)	
	Non-binary	0 (0%)	
**Basic educational qualification**	Bachelor’s degree (Before DM 509/99)	8 (35%)	
	Bachelor’s degree	15 (65%)	
	At least one first-level postgraduate degree	5 (22%)	
	At least one continuing higher education course	3 (13%)	
	No postbaccalaureate degree	12 (52%)	
	Postgraduate degree + advanced continuing education course	2 (9%)	
**Years working in hemodialysis**	1–5 y	10 (44%)	10.61 (8.73)
	6–10 y	3 (13%)	
	11–15 y	3 (13%)	
	16–20 y	2 (9%)	
	21–25 y	5 (21%)	
**Years working as a nurse**	1–10 y	6 (26%)	18.04 (9.80)
	11–20 y	8 (35%)	
	21–30 y	7 (30%)	
	31–40 y	2 (9%)	

**Table 2:** Caring Nurse–Patient Interaction Scale.

	Number of Nurses (%)					
Item	Not at All Competent 1	A Little Competent 2	Moderately Competent 3	A Lot Competent 4	Extremely Competent 5	Mean (SD)
**A—Clinical Care**						
Know how to give the treatments	0 (0%)	0 (0%)	4 (18%)	10 (43%)	9 (39%)	4.22 (0.74)
Know how to operate specialized equipment	0 (0%)	0 (0%)	4 (17%)	10 (43%)	9 (40%)	4.22 (0.74)
Check if their medications soothe their symptoms	0 (0%)	0 (0%)	1 (4%)	11 (48%)	11 (48%)	4.43 (0.59)
Give them indications and means to treat or prevent certain side effects of their medications or treatments	0 (0%)	1 (4%)	7 (30%)	8 (36%)	7 (30%)	3.91 (0.90)
Know what to do in situations where one must act quickly	0 (0%)	2 (9%)	3 (13%)	9 (39%)	9 (39%)	4.09 (0.95)
Help them with the care they cannot administer themselves	0 (0%)	0 (0%)	6 (26%)	12 (52%)	5 (22%)	3.96 (0.71)
Show ability and skill in my way of intervening with them	0 (0%)	1 (4%)	4 (17%)	10 (44%)	8 (35%)	4.09 (0.85)
Closely monitor their health condition	0 (0%)	0 (0%)	1 (4%)	16 (70%)	6 (26%)	4.22 (0.52)
Provide them with the opportunity to practice self-administered care	0 (0%)	2 (9%)	6 (26%)	11 (48%)	4 (17%)	3.74 (0.86)
**B—Relational Care**						
Help them to look for a certain equilibrium/balance in their life	0 (0%)	2 (9%)	9 (39%)	11 (48%)	1 (4%)	3.48 (0.73)
Help them to explore what is important in their life	0 (0%)	3 (13%)	10 (43%)	8 (35%)	2 (9%)	3.39 (0.84)
Help them to clarify which things they would like significant persons to bring them	0 (0%)	2 (9%)	14 (61%)	5 (21%)	2 (9%)	3.30 (0.76)
Help them to explore the meaning that they give to their health condition	0 (0%)	3 (13%)	7 (30%)	10 (44%)	3 (13%)	3.57 (0.90)
Help them to recognize the means to efficiently solve their problems	0 (0%)	1 (4%)	9 (39%)	11 (48%)	2 (9%)	3.61 (0.72)
Help them to see things from a different point of view	0 (0%)	1 (4%)	6 (26%)	14 (61%)	2 (9%)	3.74 (0.69)
Try to identify with them the consequences of their behavior	0 (0%)	0 (0%)	7 (31%)	11 (48%)	5 (22%)	3.91 (0.73)
**C—Humanistic Care**						
Treat them as complete individuals, show that I am interested in more than their health problem	0 (0%)	0 (0%)	4 (17%)	10 (44%)	9 (39%)	4.22 (0.74)
Encourage them to be hopeful, when it was appropriate	0 (0%)	0 (0%)	7 (30%)	11 (48%)	5 (22%)	3.91 (0.73)
Emphasize their efforts	0 (0%)	0 (0%)	3 (13%)	15 (65%)	5 (22%)	4.09 (0.60)
Do not have a scandalizing behavior	0 (0%)	1 (4%)	4 (18%)	12 (52%)	6 (26%)	4.00 (0.80)
**D—Comforting Care**						
Respect their privacy	0 (0%)	0 (0%)	3 (12%)	10 (44%)	10 (44%)	4.30 (0.70)
Take their basic needs into account	0 (0%)	0 (0%)	4 (17%)	9 (39%)	10 (44%)	4.26 (0.75)
Do treatments or give medications at the scheduled time	0 (0%)	0 (0%)	1 (4%)	10 (44%)	12 (52%)	4.48 (0.59)

**Table 3:** Results of the construct validity testing.

	Sociodemographic Data					
	Age	Gender	Basic Educational Qualification	Post-Basic Educational Qualification	Years Working in Hemodialysis	Years Working as a Nurse
	Correlation Coefficient (*p* Value)					
Caring Nurse–Patient Interaction Scale						
**A—Clinical Care**						
Know how to give the treatments	-	-	-	0.37 (0.085)	-	-
Know how to operate specialized equipment	-	-	-	-	0.55 (0.006)	-
Give them indications and means to treat or prevent certain side effects of their medications or treatments	0.39 (0.059)	-	-	0.40 (0.057)	-	-
Know what to do in situations where one must act quickly	0.38 (0.074)	-	-	-	0.57 (0.004)	0.43 (0.041)
Show ability and skill in my way of intervening with them	-	-	-	-	0.41 (0.052)	-
Closely monitor their health condition	-	-	-	0.48 (0.02)	-	-
Provide them with the opportunity to practice self-administered care	-	-	-	0.40 (0.055)	-	-
**B—Relational Care**						
Help them to recognize the means to efficiently solve their problems	-	0.39 (0.058)	-	-	-	-
Help them to see things from a different point of view	-	0.40 (0.056)	-	-	-	-

**Table 4:** Qualitative descriptive analysis.

Category	Descriptive Label	Recurrence
**(1) What do you think your patients expect from the nurses who care for them?**		
Professional skills	Expertise	8
	Technical skills	3
	Professionalism	2
	Highest appreciation	1
Relational skills	Caring for the relationship	2
	Identify yourself	1
	Considering patients	1
	Understand the relevance of patients’ requests	1
	Caring during the time of care	1
Caring for the person	Meeting health needs	2
	Complete satisfaction	1
	Receiving the best possible care	1
	Caring for the person primarily	1
	Treating the disease secondarily	1
	Accompaniment in the care pathway	1
Elements of the relationship	Empathy	4
	Understanding	4
	Trust	4
	Listening	2
	Reassurance	2
	Availability	2
	Kindness	2
	Nonjudgmental attitude	1
	Dialogue	1
	Respect	1
	Education	1
	Humanity	1
Dehumanization	Being indulged	1
	Complacency	1
	Subservience	1
	Professional automatism	1
**(2) Do you believe that the therapeutic nurse–patient relationship is important and/or relevant in your work?**		
Important element of quality of work	Important	16
	Empathetic relationship improves quality of work	1
Human relationship characterizes the professional	To be a nurse is to be human	1
	Lasting relationship based on trust	1
	Lasting relationship based on mutual respect	1
	Primary interpersonal skills	1
	Secondary technical skills	1
Supports nurses and patients in the care process	Supports nurses during treatments	1
	Supports patients	1
	Achieves health goals	1
	Provides confidence to the nurse	1
	Increases engagement in health education	1
	Supports dialysis with mutual understanding and respect	1
Fundamental in chronicity	Important in the care of chronic patients	1
	Essential in chronic care	1
Conditional on reciprocity	Relevant if you present critical sense and listening to each other	1
**(3) Do you believe that the nurse–patient relationship can also affect you as a person and/or your personal satisfaction?**		
Professional and personal satisfaction proportional	Much professional satisfaction	1
	Very much personal satisfaction	1
	Job satisfaction proportional to personal satisfaction	1
Feeling professionally involved	Yes	11
	Gratification	1
	Feeling appreciated	1
	Rejoicing over professional achievements	1
	Crying over complications	1
	Central professional aspect	1
Feeling personally involved	Lasting intimate personal relationship	1
	Meeting the patient’s needs satisfies professionally	1
	Empathic relationship complements specialized skills	1
	Gratification in providing well-being	1
	Getting attached to chronic patients	1
	Tightening the relationship by providing happiness	1
Fluctuating satisfaction	Sometimes/quite a bit	2
	Occasional professional gratification	1
Dehumanization	Hearing without listening	1

## Data Availability

The data presented in this study are available on request from the corresponding author. The data are not publicly available due to privacy issues.

## Public Involvement Statement

There was no public involvement in any aspect of this research.

## Guidelines and Standards Statement

This study is compliant with the Strengthening the Reporting of Observational Studies in Epidemiology (STROBE) reporting guidelines for observational studies.
